# Remembrance of things past: Towards a life-course biology of aging

**DOI:** 10.1371/journal.pbio.3003794

**Published:** 2026-05-26

**Authors:** Sara Alam, Linda Partridge, Nazif Alic

**Affiliations:** Institute of Healthy Ageing and the Research Department of Genetics, Evolution, and Environment, University College London, London, United Kingdom; Harvard University T H Chan School of Public Health, UNITED STATES OF AMERICA

## Abstract

Globally, the growing proportion of older individuals is imposing personal and societal costs. However, interventions that slow aging are possible; for example, dampened nutrient signaling pathway activity in animal models promotes better health later in life. Recent findings indicate that such interventions have long-term effects even when applied transiently in early adulthood, forming a “physiological memory.” Similar memory has been extensively documented in human epidemiology, where the health of older people is shaped by their earlier environmental exposures, such as diet composition. This Essay argues that the study of the biology of aging should encompass determinants of healthspan across the entire life course.

## Abstract 2

In the last few years, evidence has emerged from the basic biology of ageing suggesting that ageing can be influenced by transient conditions experienced earlier in life. The relevance of these finding is supported by decades of research in human epidemiology and disease-focused experimental studies. Here, we summarise the key findings to propose the ageing field take into consideration the whole life-course of an individual to understand the plasticity of late-life health and longevity.

## Introduction

Age‌‌ is the biggest risk factor for most diseases, including high-impact conditions such as cancer, cardiovascular disease, and neurodegenerative disease [1]. The proportion of aged individuals is increasing globally, and this is resulting in economic, healthcare, and societal strains. Consequently, there is a heightened need to understand the aging process to ensure better health in older age [2]. Aging has been examined from a number of perspectives, ranging from evolutionary biology to human epidemiology and, more recently, basic biology. It is the study of the latter, the biology of aging, that currently holds the potential to generate novel treatments. In this Essay, we argue that research into the basic biology of aging needs to adapt concepts and approaches from human life-course epidemiology in order to adequately address the needs of numerous, diverse, aging populations.

## Nutrient signaling and aging

**Fig 1:**
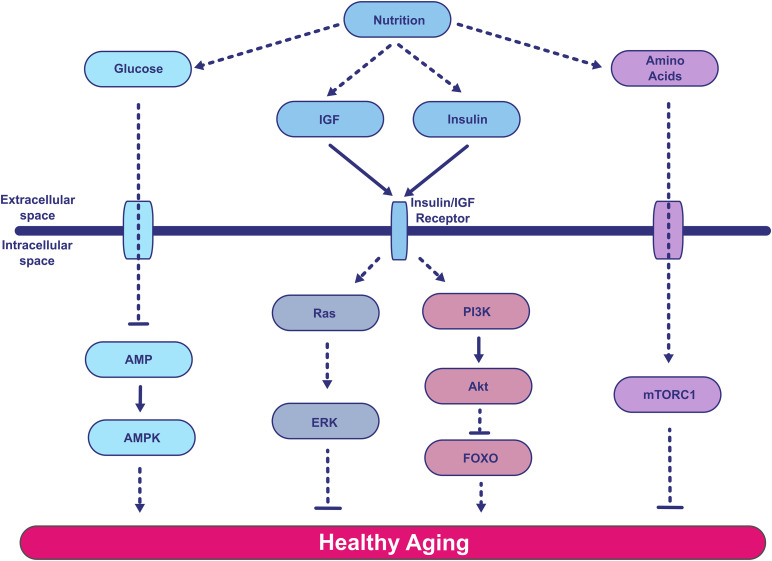
The impact of nutrient signaling on aging. A simplified summary of some of the known nutrient signaling pathways, including insulin/IGF, mTORC1, ERK, and AMPK, and how they broadly impact healthy aging. This diagram is based on previously published schematics, omitting pathway cross-regulation for clarity [12–14]. Dotted arrows denote parts of pathways with missing components for simplification. AMPK, AMP-activated protein kinase; ERK, extracellular signal-regulated kinase; FOXO, Forkhead box O; IGF, insulin-like growth factor; mTORC1, mechanistic target of rapamycin complex 1; PI3K, phosphoinositide 3-kinase.

The past four decades have seen a profound shift in how we view aging, from seeing age as a non-modifiable risk factor for many diseases, to understanding aging as a plastic process that can be targeted for improved health in older age. The study of the mechanisms behind aging and its plasticity has, since the beginning, been tightly linked to nutrient signaling pathways (Fig 1). A classic example is the significant extension of *Caenorhabditis elegans* life span as a consequence of reduced activity of *age-1* or *daf-2*, two genes that encode core components of the nutrient-responsive insulin/insulin-like growth factor (IGF) signaling (IIS) pathway [3–5]. IIS is a neuroendocrine signaling network defined by the structure of the extracellular ligands (which resemble insulin) and their cognate cell-surface receptors (which are related to the insulin receptor). Experimental evidence has accumulated over decades demonstrating that reduced IIS activity can decelerate aging. This effect is strikingly well conserved across large evolutionary distances, and is observed in worms, flies, and mice. Indeed, genetic epidemiology studies indicate that at least some components of this network affect human aging. For example, *FOXO3A* is one of the few genes that have been robustly associated with human longevity [6–9]. *FOXO3A* encodes one of the members of the Forkhead box O (FoxO) transcription factor family in humans, which seems to be at the core of the IIS pathway’s effects on longevity [4,10,11]. This suggests that IIS manipulation is a promising way to slow human aging.

Aging is marked by the degeneration of several processes, including proteostasis, mitochondrial quality control and activity, metabolic and immune function, and other interconnected aspects of cellular and physiological function, altogether comprising the “hallmarks of aging” [15]. Importantly, several of these hallmarks are targeted in concert by direct modulation of IIS, and are collectively responsible for the substantial changes in the rate of aging under IIS-reduced conditions (Fig 1) [16–20]. The responses are tissue-specific, often coordinated by a number of downstream transcription factors and other effectors that act in a tissue-restricted manner [18,21–24]. Conversely, changing the intracellular IIS activity in specific tissues and cell types is sufficient to extend life span of the whole organism [25–28].

The IIS pathway does not act alone, but is integrated with several others, at both the endocrine and intracellular level, all of which coordinately control animal physiology (Fig 1). For example, IGF1 production can be regulated though the somatotropic axis in mammals to mediate part of the effects of growth hormone (GH) [29]. Inside the cell, receptor activation may result in the activation of both the Akt and extracellular signal-regulated kinase (ERK) pathways, which in turn interact with other intracellular nutrient- and energy-signaling pathways such as those centered around mechanistic target of rapamycin complexes 1 and 2 (mTORC1 and mTORC2) and AMP-activated protein kinase (AMPK) [30]. There is evidence that these pathways also drive aging; for example, GH may have key importance in mammals [31], and ERK has been documented to be relevant in flies, mice, and potentially in humans [23,32–34]. Several of these components can be targeted by drugs to induce a longer and healthier life span: mTORC1 can be targeted by rapamycin‌‌ [35], ERK by trametinib [32], and AMPK by metformin [36]. Interestingly, endocrine signals coordinately regulate the activity of these intracellular pathways, and the interventions that target these upstream signals often provide the largest and most robust effects; examples include targeting *Drosophila* insulin-like peptides in flies, or GH in mammals [31,37,38]. Viewed from this perspective, it is unsurprising that combination drug treatments aimed at kinases that would be coordinately regulated downstream of the endocrine signal(s) can have substantially additive effects on life span [34].

Once the components of the network started being explored as drug targets, and the questions of when treatments could be effective were raised, some curious observations emerged. For example, transient rapamycin treatment in mice and fruit flies has a long-term beneficial effect; notably, these long-term effects of transient rapamycin treatment could not be explained by prolonged mTORC1 inhibition [27,39]. Similarly, short-term treatment with an mTORC1 inhibitor in humans, aside from improving vaccine efficacy in older individuals, may also have a long-term beneficial effect on susceptibility to infection [40,41]. The benefits of transient treatment are seen not only for mTORC1 inhibitors: activation of the transcription factor FoxO restricted to early adulthood was sufficient to extend life span in flies [26]. Additionally, reducing early adulthood protein synthesis in *Drosophila* extends life span by lowering the levels of juvenile hormone (JH) at this stage; this could be via reducing IIS, as JH potentially mediates life span extension by IIS inhibition [42–45]. These surprising findings add to a growing body of evidence suggesting that interventions in early life, particularly those impacting nutrient signaling, can have an effect on subsequent aging. The way they do so is poorly understood; however, we can define this lasting state of the body as having “physiological memory.” This term is intentionally broad, highlighting that the animal’s physiology seems to be altered in the long term, while being agnostic as to the underlying mechanisms.

The fundamental role of the nutrient signaling network is to mediate responses of cells to nutritional environments. It has long been known that dietary regimens, such as dietary restriction and intermittent fasting, can robustly extend life span in multiple model organisms and, while the relationship is complicated and multi-factorial, nutrient signaling is believed to be at the core of how feeding regimens can slow aging [46–48]. Recent findings have shown that such dietary regimens can have long-term impacts that persist after administration; late-life mortality analysis indicated that mice are affected by past diets despite a dietary change, with *ad libitum* diet leaving a stronger memory than dietary restriction [49]. Work in *C. elegans* has also shown that removal of the food source *Escherichia coli* can extend life span, even if limited to the first 10–15 days of adulthood [50]. Intermittent fasting can also extend life span when restricted to early adulthood, as seen both in *C. elegans* and in *Drosophila* [51,52]. While the exact mechanisms are yet to be identified, it was shown that in *Drosophila* they are independent of the S6K-specific branch of mTORC1 signaling, informing the direction for future work [51]. Further work on fruit flies has shown that other dietary changes restricted to development or early adulthood can still have long-term effects on life span [53–55]. For example, short-term exposure to a high sugar diet in early adulthood shortens subsequent life span in a manner dependent on *foxo*, the *Drosophila* orthologue of *FOXO3A* [54]. Similarly, the life span-extending effects of short-term methionine restriction are also dependent on FoxO-induced methionine sulfoxide reductase A expression [55].

Overall, work with model organisms clearly shows the potential of short-term alterations of nutrient signaling (pharmacological, genetic, or dietary) to slow aging. These long-term effects may be dependent on a physiological memory engrained in the system. But is this physiological memory applicable to human aging? To explore this, we will turn to some key observations in human epidemiology.

## Evidence for physiological memory in human disease epidemiology

Substantial epidemiological evidence supports the notion that the physiology and health of an aged adult are influenced not only by their current environment but also by exposure to specific insults or conditions throughout their life. Most surprising is the influence of the in utero and early-life environment on metabolic health in late adulthood, which has been documented for decades and has culminated in the developmental origins of adult disease (DOD) hypothesis. This field began with striking work by Barker and colleagues [56,57], who documented a strong negative correlation between the weight of an infant at birth and the likelihood of them developing type 2 diabetes (T2D), hypertension, and death from cardiovascular disease by 75 years of age. This suggests that the nutritional environment experienced during fetal development could influence the health of the individual decades after birth. The key idea that past environmental exposures can contribute to health outcomes in late adulthood took shape in a life-course approach to epidemiology [58], which spurred on research into the underlying biological mechanisms, as well as societal implications, with a focus on metabolic health.

The correlations between in utero nutrition and late-life metabolic health have been demonstrated in several other longitudinal studies, many of which make use of exceptional historical events that exposed pregnant mothers to abnormal conditions for a specific amount of time. For example, in the winter and spring of 1944, the Netherlands experienced a famine, and among the children of mothers who were pregnant during this period there was a strong correlation between the restriction of calories in utero and either increased or decreased likelihood of late-life obesity and cardiovascular disease, depending on whether the famine occurred at early or late gestation, respectively [59]. These correlations were reported in multiple other studies following individuals who experienced famines across the globe [60–64]. Longitudinal studies following the effects of extreme nutritional events are not limited to famines; between 1940 and 1953, sugar was rationed in the United Kingdom, limiting consumption to the current daily recommended amounts. Exposure to sugar rationing in utero significantly reduced the likelihood of developing hypertension or T2D, and this effect was intensified if rationing continued up to 12 months after birth [65,66]. These remarkable correlations were, importantly, replicable in controlled animal studies, strengthening the conclusion that late-life metabolic health can be affected by developmental nutritional exposures [67,68].

The DOD theory establishes that a physiological memory can be formed during development and impact health in the long term. However, information on adults that have experienced a short-term but profound metabolic change, or have undergone interventions that alter metabolic health, is not as extensive. Nevertheless, a few studies have tracked the long-term effects of diabetes or obesity, even when these conditions are controlled or reversed, identifying a way in which short-term metabolic dysfunction can be cemented in what has been termed “metabolic memory” (which we suggest to be a specific instance of a broader phenomenon of physiological memory). Such a metabolic memory formed in adulthood is seen in individuals with obesity who lose weight: these individuals often experience weight re-gain (often termed a “yo-yo” effect) [69,70], which is likely driven by long-term and irreversible physiological changes caused by obesity. Using single-cell sequencing approaches, Hinte and colleagues recently found that, despite weight loss, adipose tissue from previously obese humans and mice seemingly retains a transcriptional memory of the metabolic state of obesity, as well as persistent pro-inflammatory signatures and impaired adipocyte functionality and metabolism, which potentially primes individuals who previously had obesity to weight re-gain [71]. This obesity-induced and post-weight-loss-retained pro-inflammatory phenotype, as well as insulin insensitivity, has also been observed in other studies, and could not be resolved by preventing hyperphagia, indicating that the yo-yo effect may indeed be caused by a physiological memory of obesity [72]. Additional evidence of such memory, including metabolic memory of T2D despite glucose level correction, has been thoroughly reviewed by others [73]. Such long-term maintenance of pro-inflammatory phenotypes or IIS disruption would be expected to influence aging significantly [74].

While there is a tight link between metabolic disease and aging, tracking the formation and persistence of metabolic or broader physiological memory in individuals that have a healthy early adulthood is key to extend this concept to the broad treatment or prevention of aging. One such work will be the legacy study of the CALERIE phase 2 trial [75], where healthy, middle-aged individuals without obesity experienced moderate caloric restriction for 2 years, with positive outcomes, such as improvements in cardiometabolic health markers [76]. Ten to fifteen years following the end of treatment, participants will be reassessed to monitor the long-term effects of this short-term intervention [77]. Results from this legacy study may reveal whether physiological memory can be formed in healthy individuals, and whether this can meaningfully impact health and aging in the long term.

Altogether, there is abundant evidence suggesting that humans can retain a physiological memory, including that of past diets and of the metabolic states they lead to. This tallies well with the recent observation of long-term effects on aging from manipulations of nutrition or of nutrient signaling pathways in model organisms, and strongly suggests that early-life environments can have a profound effect on the biology of human aging, regardless of how transiently they are experienced. But how is this memory formed and retained?

## Possible mechanisms of physiological memory

Nutrient signaling pathways, and other similar regulators of animal physiology, are thought to act in a homeostatic manner, providing continuous adjustments to maintain function in a changing internal and external environment. Their responses and actions are essentially thought of as short-term, so it is important to understand how they are able to cause a long-term change in the rate of aging.

One major contender is the epigenome, including but not limited to DNA methylation and histone modifications. Indeed, it is becoming increasingly clear that nutrient signaling and metabolism have a complex and significant impact on epigenetic remodeling [78,79]. Epigenetic modifications can affect the expression of large sets of genes, enabling metabolic pathways to be more robustly manipulated than if individual genes are targeted alone. Additionally, epigenetic modifications have staying power, which can be carried across generations of cell division [80] and sexual reproduction. Indeed, transgenerational inheritance has been demonstrated in multiple model systems, where parental epigenetic changes are transferrable and impact progeny aging over multiple generations [81–84]. Importantly, there is ample evidence that nutrient signaling can alter the epigenome. For example, fasting leads to chromatin remodeling via mTORC1 and RNA polymerase I inhibition [85], and AMPK directly phosphorylates histone H2B, which facilitates the expression of AMPK-responsive genes [86]. While there is still much to be explored, nutrient signaling can both directly and indirectly alter the epigenome [87–94], thus giving these pathways the potential to have lasting effects beyond their activation or inhibition.

Epigenetic changes formed during development could explain the DOD phenomenon [95,96]. More recent work, in the context of metabolic disease, supports the idea that this form of physiological memory can also be generated in adulthood [97]. For example, the difference in H3K4me3, H3K27me3, H3K27ac, and H3K4me1 signatures seen between adipocytes from animals with obesity compared with lean animals is partially maintained even after weight loss, and may explain the predisposition of mice that have previously had obesity to regaining weight [71]. Furthermore, there is substantial evidence that epigenetic regulation underlies metabolic memory in other contexts as well (reviewed previously [73]). Importantly, mounting evidence shows that the epigenome can strongly impact longevity [78,93,98]. Hence, it is possible that the epigenetic mechanisms demonstrated to underlie metabolic memory are the same as those underlying other forms of physiological memory that can affect aging. Indeed, epigenetic changes that affect gene accessibility seem to hold memory of life span-extending interventions [26,99]. The memory effect of FoxO activation in *Drosophila* early adulthood, which can extend life span significantly, seems to be dependent on the presence of functional switch/sucrose non-fermentable (SWI/SNF) and imitation SWI (ISWI) complexes, which are responsible for opening chromatin and enabling transcription of target genes [26].

Additional hypotheses implicating epigenetic modifications as the source of physiological memory can be proposed to explain other recent findings. For example, it is possible that the life span-extending effects of methionine restriction in early adulthood, which upregulates levels of the methyl-group donor S-adenosyl methionine (SAM), can be mediated by long-term changes in histone methylation [55,100,101].

Epigenetically encoded physiological memory need not be the only mechanism by which early-life events can influence longevity. On a cellular level, post-translational modifications and behaviors of proteins act as a short-term as well as long-term and *trans-*generational memory store. Mnemons, or protein condensates, occur in multicellular organisms, and have been thoroughly studied in *Saccharomyces cerevisiae*, enabling cells to remember mating cues, stressors, or nutritional environments. Many such protein condensates are RNA-binding proteins, suggesting that the formation of condensates can have broad and long-lasting effects on the translation of specific proteins [102]. Moreover, aging may be partially caused by the accumulation of irreversible damage, which can become a physiological memory. For example, damage by advanced glycation end-products and reactive oxygen species (ROS) can begin in early adulthood and lead to long-term changes that impact longevity [103,104]. Exposure to inflammation, particularly chronically, can also cause cumulative damage that can accelerate aging [105]. Additionally, the gut microbiome, which is tightly linked to the progression of aging, can have its composition altered in the long term by dietary conditions experienced in the past, suggesting that it may hold part of the whole body’s physiological memory [106–108].

Is the physiological memory that impacts whole-body aging held in a specific tissue? Identifying its location could be key to effective manipulation. The adipose tissue is consistently identified as a source of physiological memory in multiple organisms, including humans [71]. FoxO activation in the *Drosophila* fat body alone was sufficient to extend life span, even when restricted to early adulthood, and while this organ can also function as a hepatic tissue, the liver does not seem to be responsible for physiological memory in mice [26,49,109]. Additionally, memory may be retained in the adipose tissue through changes in its composition. High sugar or fat diets, diabetes, and obesity, even in the short term, can affect the immune cell content and the inflammation of this tissue, forming a potential physiological memory that can impact whole-body aging [71,110–112]. The gut may also be responsible for the maintenance of physiological memory, as it is in *Drosophila*. Here, the memory of rapamycin treatment seems to be held in a manner dependent on the upregulation of lysosomal α-mannosidase V expression and persistent induction of autophagy [27]. While individual tissues may be important, it is entirely possible that robust longevity and healthspan are dependent on the holistic maintenance of good health in multiple organs, with a memory potentially encoded in each tissue having an important role.

Additionally, vital to understanding physiological memory is knowing when it is possible to cement it in the body. For example, while the correlation mentioned earlier between exposure to sugar rationing and metabolic health is clear, if children experienced rationing for longer than 12 months after birth, there was no additional protection against developing T2D or hypertension, indicating that a significant reduction in formation of metabolic memory occurs after the first year [65,66]. This perhaps indicates a reduction in the system’s plasticity, despite the body still developing. Similarly, ROS have different effects on life span depending on when they are modulated: in *C. elegans*, even just a two-day difference in age can affect whether an antioxidant has a positive or negligible effect on life span [113]. If we consider an animal’s entire life course, when is the system most plastic and able to shape aging? And at which points is any memory formed still reversible? The answers to these questions may be context dependent: the effects of life span-extending or -shortening events may depend on when the relevant biological processes take place. An example of this comes from work in *Drosophila* showing that reducing protein synthesis in early adulthood alone extends life span, possibly because this is when translation is at its peak in fruit flies [45].

Much work has yet to be done to truly understand the core mechanisms behind physiological memory, and only a handful of studies have functionally linked these molecular mechanisms to longevity. This is in part because the biology of aging, as a research discipline, has yet to pay attention to the untapped potential of studying aging from a life-course perspective.

## Towards a life-course understanding of the biology of aging

Understanding how physiological memory is formed and the consequences it has for health in older age should be a key part of understanding the biology of human aging, for several reasons. First, given the growing global obesity epidemic affecting multiple generations, and the current view that obesity accelerates aging, it is very likely that nutrition and dietary habits throughout life are having and will continue to have a significant impact on how we age [66,114–116]. Importantly, metabolic programing arising from past dietary habits and metabolic disease may persist regardless of whether a healthy metabolic state is eventually achieved [71]. Hence, unless strategies are specifically developed to combat this programming, the effects of metabolic disease on the aged population will linger, despite our best efforts. Second, the diversity in nutritional and other environments that individuals and populations are exposed to during their lifetime is highly likely to lead to disparities, often linked to socioeconomic status, which could affect late-life health and aging [117–120]. Are particular life experiences going to make some interventions targeting aging ineffective in certain human populations?

Additionally, a detailed mechanistic understanding of the points in the life course that can profoundly shape the biology of aging may open up avenues for potentially easy, effective, cheap, and broad prophylactic treatments. Indeed, there is mounting evidence that pharmacological interventions that target aging may not have the same efficacy across the life course [121]. Knowing when they are effective may alleviate not only the unwanted effects of pharmaceuticals but also the economic burden they impose if administered chronically. Dietary, lifestyle, and behavioral interventions that may extend life span are also unlikely to be maintained for a lifetime, and their implementation as well as efficacy may vary drastically between people in different socio-economic circumstances [122]. Thus, a biological understanding of when they are most effective, and in which individuals, will help improve impact and minimize costs. Furthermore, there is the possibility of targeting physiological memory in later life, either by reversing physiological memory that would negatively impact life span, or mimicking that which would lead to healthy aging.

**Fig 2:**
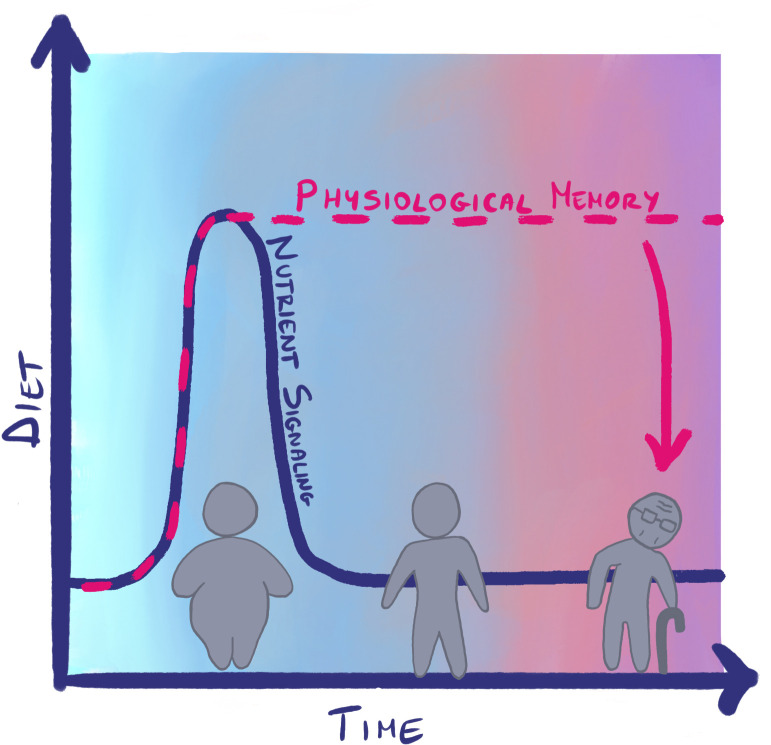
A life-course dimension to the mechanism of aging. Temporal changes in environmental exposures, such as diet, can lead to relatively transient modulation in the activity of pathways that promote aging, such as nutrient signaling pathways. This can be sufficient to form physiological memory and impact subsequent aging, health, and longevity.

We think that nutrient signaling pathways are likely to have an important role in programing animal physiology towards particular aging outcomes. This is because we are starting to see evidence that they can program life span and older-age health in animal models; because they respond to environmental cues that we know from epidemiological studies to be relevant to programing of human aging and age-related diseases; and because they provide a plausible mechanistic link between past environments and health in older age, for example, by their known ability to alter the epigenome. The aging field has identified multiple, highly interconnected nutrient signaling pathways that independently impact the rate of aging, modulating a number of cellular and physiological processes in a number of tissues and organs. Now, to the many diagrams summarizing the mechanisms of how these pathways act on aging, the temporal dimension of physiological memory should be introduced (Fig 2). In essence, we propose that to understand animal aging, we need to move towards a more dynamic understanding of the biological mechanisms whereby different environmental exposures, internal processes, and longevity-promoting mechanisms integrate across the life-course in model organisms.

How is physiological memory formed and retained in an animal? What kind of nutritional and signaling states can be imprinted? Are there points along an animal’s life course when memory is most readily formed and retained? Can the understanding of physiological memory be used to tailor anti-aging therapy? We believe answering these questions will be key to advancing our understanding of biogerontology, and will require the targeted efforts of researchers interested in the biology of aging.

## Abbreviations

AMPK: AMP-activated protein kinase
DOD: developmental origins of adult disease
ERK: extracellular signal-regulated kinase
FoxO: Forkhead box O
GH: growth hormone
IGF: insulin-like growth factor
ISWI: imitation SWI
JH: juvenile hormone
ROS: reactive oxygen species
SAM: S-adenosyl methionine
SWI/SNF: switch/sucrose non-fermentable
T2D: type‌‌ 2 diabetes

## References

[1] Kuan V, Fraser HC, Hingorani M, Denaxas S, Gonzalez-Izquierdo A, Direk K. Data-driven identification of ageing-related diseases from electronic health records. Sci Rep. 2021;11:2938. doi: 10.1038/s41598-021-82459-y 33536532 PMC7859412

[2] Partridge L, Deelen J, Slagboom PE. Facing up to the global challenges of ageing. Nature. 2018;561(7721):45–56. doi: 10.1038/s41586-018-0457-8 30185958

[3] Friedman DB, Johnson TE. A mutation in the age-1 gene in *Caenorhabditis elegans* lengthens life and reduces hermaphrodite fertility. Genetics. 1988;118(1):75–86. doi: 10.1093/genetics/118.1.75 8608934 PMC1203268

[4] Kenyon C, Chang J, Gensch E, Rudner A, Tabtiang R. A *C. elegans* mutant that lives twice as long as wild type. Nature. 1993;366(6454):461–4. doi: 10.1038/366461a0 8247153

[5] Kolb H, Kempf K, Martin S. Insulin and aging - a disappointing relationship. Front Endocrinol (Lausanne). 2023;14:1261298. doi: 10.3389/fendo.2023.1261298 37854186 PMC10579801

[6] Willcox BJ, Donlon TA, He Q, Chen R, Grove JS, Yano K, et al. FOXO3A genotype is strongly associated with human longevity. Proc Natl Acad Sci U S A. 2008;105(37):13987–92. doi: 10.1073/pnas.0801030105 18765803 PMC2544566

[7] Pawlikowska L, Hu D, Huntsman S, Sung A, Chu C, Chen J, et al. Association of common genetic variation in the insulin/IGF1 signaling pathway with human longevity. Aging Cell. 2009;8(4):460–72. doi: 10.1111/j.1474-9726.2009.00493.x 19489743 PMC3652804

[8] Broer L, Buchman AS, Deelen J, Evans DS, Faul JD, Lunetta KL, et al. GWAS of longevity in CHARGE consortium confirms APOE and FOXO3 candidacy. J Gerontol A Biol Sci Med Sci. 2015;70(1):110–8. doi: 10.1093/gerona/glu166 25199915 PMC4296168

[9] Deelen J, Evans DS, Arking DE, Tesi N, Nygaard M, Liu X, et al. A meta-analysis of genome-wide association studies identifies multiple longevity genes. Nat Commun. 2019;10(1):3669. doi: 10.1038/s41467-019-11558-2 31413261 PMC6694136

[10] Slack C, Giannakou ME, Foley A, Goss M, Partridge L. dFOXO-independent effects of reduced insulin-like signaling in *Drosophila*. Aging Cell. 2011;10(5):735–48. doi: 10.1111/j.1474-9726.2011.00707.x 21443682 PMC3193374

[11] Yamamoto R, Tatar M. Insulin receptor substrate chico acts with the transcription factor FOXO to extend *Drosophila* lifespan. Aging Cell. 2011;10(4):729–32. doi: 10.1111/j.1474-9726.2011.00716.x 21518241 PMC3135774

[12] Bettedi L, Foukas LC. Growth factor, energy and nutrient sensing signalling pathways in metabolic ageing. Biogerontology. 2017;18(6):913–29. doi: 10.1007/s10522-017-9724-6 28795262 PMC5684302

[13] Green CL, Lamming DW, Fontana L. Molecular mechanisms of dietary restriction promoting health and longevity. Nat Rev Mol Cell Biol. 2022;23(1):56–73. doi: 10.1038/s41580-021-00411-4 34518687 PMC8692439

[14] Alic N, Partridge L. Death and dessert: nutrient signalling pathways and ageing. Curr Opin Cell Biol. 2011;23(6):738–43. doi: 10.1016/j.ceb.2011.07.006 21835601 PMC4335171

[15] López-Otín C, Blasco MA, Partridge L, Serrano M, Kroemer G. Hallmarks of aging: an expanding universe. Cell. 2023;186(2):243–78. doi: 10.1016/j.cell.2022.11.001 36599349

[16] Palikaras K, Lionaki E, Tavernarakis N. Coordination of mitophagy and mitochondrial biogenesis during ageing in *C. elegans*. Nature. 2015;521(7553):525–8. doi: 10.1038/nature14300 25896323

[17] McCormack S, Yadav S, Shokal U, Kenney E, Cooper D, Eleftherianos I. The insulin receptor substrate Chico regulates antibacterial immune function in *Drosophila*. Immun Ageing. 2016;13:15. doi: 10.1186/s12979-016-0072-1 27134635 PMC4852101

[18] Tain LS, Sehlke R, Jain C, Chokkalingam M, Nagaraj N, Essers P, et al. A proteomic atlas of insulin signalling reveals tissue-specific mechanisms of longevity assurance. Mol Syst Biol. 2017;13(9):939. doi: 10.15252/msb.20177663 28916541 PMC5615923

[19] Duxbury EML, Carlsson H, Kimberley A, Ridge Y, Johnson K, Maklakov AA. Reduced insulin/IGF-1 signalling upregulates two anti-viral immune pathways, decreases viral load and increases survival under viral infection in *C. elegans*. Geroscience. 2024;46(6):5767–80. doi: 10.1007/s11357-024-01147-7 38589671 PMC11493891

[20] Molière A, Park JYC, Goyala A, Vayndorf EM, Zhang B, Hsiung KC, et al. Improved resilience and proteostasis mediate longevity upon DAF-2 degradation in old age. Geroscience. 2024;46(5):5015–36. doi: 10.1007/s11357-024-01232-x 38900346 PMC11335714

[21] Tain LS, Sehlke R, Meilenbrock RL, Leech T, Paulitz J, Chokkalingam M. Tissue-specific modulation of gene expression in response to lowered insulin signalling in *Drosophila*. Elife. 2021. doi: 10.7554/eLife.67275 33879316 PMC8060030

[22] Malik Y, Goncalves Silva I, Diazgranados RR, Selman C, Alic N, Tullet JM. Timing of TORC1 inhibition dictates Pol III involvement in *Caenorhabditis elegans* longevity. Life Sci Alliance. 2024;7(7):e202402735. doi: 10.26508/lsa.202402735 38740431 PMC11091362

[23] Ureña E, Xu B, Regan JC, Atilano ML, Minkley LJ, Filer D. Trametinib ameliorates aging-associated gut pathology in Drosophila females by reducing Pol III activity in intestinal stem cells. Proc Natl Acad Sci U S A. 2024;121(4):e2311313121. doi: 10.1073/pnas.2311313121 38241436 PMC10823232

[24] Kivimäki M, Frank P, Pentti J, Jokela M, Nyberg ST, Blake A, et al. Proteomic organ-specific ageing signatures and 20-year risk of age-related diseases: the Whitehall II observational cohort study. Lancet Digit Health. 2025;7(3):e195–204. doi: 10.1016/j.landig.2025.01.006 40015764

[25] Libina N, Berman JR, Kenyon C. Tissue-specific activities of *C. elegans* DAF-16 in the regulation of lifespan. Cell. 2003;115(4):489–502. doi: 10.1016/s0092-8674(03)00889-4 14622602

[26] Martínez Corrales G, Li M, Svermova T, Goncalves A, Voicu D, Dobson AJ, et al. Transcriptional memory of dFOXO activation in youth curtails later-life mortality through chromatin remodeling and Xbp1. Nat Aging. 2022;2(12):1176–90. doi: 10.1038/s43587-022-00312-x 37118537 PMC7614430

[27] Juricic P, Lu Y-X, Leech T, Drews LF, Paulitz J, Lu J, et al. Long-lasting geroprotection from brief rapamycin treatment in early adulthood by persistently increased intestinal autophagy. Nat Aging. 2022;2(9):824–36. doi: 10.1038/s43587-022-00278-w 37118497 PMC10154223

[28] Li M, Shou H, Martínez Corrales G, Svermova T, Franco AV, Alic N. Xbp1 targets canonical UPRER and non-canonical pathways in separate tissues to promote longevity. iScience. 2024;27(6):109962. doi: 10.1016/j.isci.2024.109962 38832022 PMC11144730

[29] Al-Samerria S, Radovick S. The role of insulin-like growth factor-1 (IGF-1) in the control of neuroendocrine regulation of growth. Cells. 2021;10(10):2664. doi: 10.3390/cells10102664 34685644 PMC8534318

[30] Selman C, Partridge L, Withers DJ. Replication of extended lifespan phenotype in mice with deletion of insulin receptor substrate 1. PLoS One. 2011;6(1):e16144. doi: 10.1371/journal.pone.0016144 21283571 PMC3026792

[31] Brown-Borg HM. Growth hormone, not IGF-1 is the key longevity regulator in mammals. J Gerontol A Biol Sci Med Sci. 2022 Apr 18;77(9):1719–23. doi: 10.1093/gerona/glac092 35436323 PMC9434454

[32] Slack C, Alic N, Foley A, Cabecinha M, Hoddinott MP, Partridge L. The Ras-Erk-ETS-signaling pathway is a drug target for longevity. Cell. 2015;162(1):72–83. doi: 10.1016/j.cell.2015.06.023 26119340 PMC4518474

[33] Baghdadi M, Hinterding H, Gehrmann T, Putter P, Neuerburg M, Lakenberg N, et al. Functional characterisation of rare variants in genes encoding the MAPK/ERK signalling pathway identified in long-lived Leiden Longevity Study participants. Geroscience. 2026;48(1):625–45. doi: 10.1007/s11357-025-01699-2 40442464 PMC12972501

[34] Gkioni L, Nespital T, Baghdadi M, Monzó C, Bali J, Nassr T, et al. The geroprotectors trametinib and rapamycin combine additively to extend mouse healthspan and lifespan. Nat Aging. 2025;5(7):1249–65. doi: 10.1038/s43587-025-00876-4 40437307 PMC12270913

[35] Harrison DE, Strong R, Sharp ZD, Nelson JF, Astle CM, Flurkey K, et al. Rapamycin fed late in life extends lifespan in genetically heterogeneous mice. Nature. 2009;460(7253):392–5. doi: 10.1038/nature08221 19587680 PMC2786175

[36] Martin-Montalvo A, Mercken EM, Mitchell SJ, Palacios HH, Mote PL, Scheibye-Knudsen M, et al. Metformin improves healthspan and lifespan in mice. Nat Commun. 2013;4:2192. doi: 10.1038/ncomms3192 23900241 PMC3736576

[37] Broughton SJ, Piper MDW, Ikeya T, Bass TM, Jacobson J, Driege Y, et al. Longer lifespan, altered metabolism, and stress resistance in *Drosophila* from ablation of cells making insulin-like ligands. Proc Natl Acad Sci U S A. 2005;102(8):3105–10. doi: 10.1073/pnas.0405775102 15708981 PMC549445

[38] Grönke S, Mildner A, Fellert S, Tennagels N, Petry S, Müller G, et al. Brummer lipase is an evolutionary conserved fat storage regulator in *Drosophila*. Cell Metab. 2005;1(5):323–30. doi: 10.1016/j.cmet.2005.04.003 16054079

[39] Bitto A, Ito TK, Pineda VV, LeTexier NJ, Huang HZ, Sutlief E, et al. Transient rapamycin treatment can increase lifespan and healthspan in middle-aged mice. Elife. 2016;5:e16351. doi: 10.7554/eLife.16351 27549339 PMC4996648

[40] Mannick JB, Morris M, Hockey H-UP, Roma G, Beibel M, Kulmatycki K, et al. TORC1 inhibition enhances immune function and reduces infections in the elderly. Sci Transl Med. 2018;10(449):eaaq1564. doi: 10.1126/scitranslmed.aaq1564 29997249

[41] Perkins GB, Tunbridge MJ, Chai CS, Hope CM, Yeow AEL, Salehi T, et al. Mechanistic target of rapamycin inhibitors and vaccine response in kidney transplant recipients. J Am Soc Nephrol. 2025;36(11):2213–27. doi: 10.1681/ASN.0000000716 40403135 PMC12591682

[42] Tatar M, Kopelman A, Epstein D, Tu MP, Yin CM, Garofalo RS. A mutant *Drosophila* insulin receptor homolog that extends life-span and impairs neuroendocrine function. Science. 2001;292(5514):107–10. doi: 10.1126/science.1057987 11292875

[43] Yamamoto R, Bai H, Dolezal AG, Amdam G, Tatar M. Juvenile hormone regulation of *Drosophila* aging. BMC Biol. 2013;11:85. doi: 10.1186/1741-7007-11-85 23866071 PMC3726347

[44] Tian Y, Garcia G, Bian Q, Steffen KK, Joe L, Wolff S, et al. Mitochondrial stress induces chromatin reorganization to promote longevity and UPR(mt). Cell. 2016;165(5):1197–208. doi: 10.1016/j.cell.2016.04.011 27133166 PMC4889216

[45] Kim HS, Parker DJ, Hardiman MM, Munkácsy E, Jiang N, Rogers AN, et al. Early-adulthood spike in protein translation drives aging via juvenile hormone/germline signaling. Nat Commun. 2023;14(1):5021. doi: 10.1038/s41467-023-40618-x 37596266 PMC10439225

[46] Hou L, Wang D, Chen D, Liu Y, Zhang Y, Cheng H, et al. A systems approach to reverse engineer lifespan extension by dietary restriction. Cell Metab. 2016;23(3):529–40. doi: 10.1016/j.cmet.2016.02.002 26959186 PMC5110149

[47] Zandveld J, van den Heuvel J, Zwaan BJ, Piper MDW. Both overlapping and independent mechanisms determine how diet and insulin-ligand knockouts extend lifespan of *Drosophila melanogaster*. NPJ Aging Mech Dis. 2017;3:4. doi: 10.1038/s41514-017-0004-0 28649422 PMC5445580

[48] Hwangbo D-S, Lee H-Y, Abozaid LS, Min K-J. Mechanisms of lifespan regulation by calorie restriction and intermittent fasting in model organisms. Nutrients. 2020;12(4):1194. doi: 10.3390/nu12041194 32344591 PMC7230387

[49] Hahn O, Drews LF, Nguyen A, Tatsuta T, Gkioni L, Hendrich O, et al. A nutritional memory effect counteracts benefits of dietary restriction in old mice. Nat Metab. 2019;1(11):1059–73. doi: 10.1038/s42255-019-0121-0 31742247 PMC6861129

[50] Lee GD, Wilson MA, Zhu M, Wolkow CA, de Cabo R, Ingram DK, et al. Dietary deprivation extends lifespan in *Caenorhabditis elegans*. Aging Cell. 2006;5(6):515–24. doi: 10.1111/j.1474-9726.2006.00241.x 17096674 PMC2546582

[51] Catterson JH, Khericha M, Dyson MC, Vincent AJ, Callard R, Haveron SM, et al. Short-term, intermittent fasting induces long-lasting gut health and tor-independent lifespan extension. Curr Biol. 2018;28(11):1714-1724.e4. doi: 10.1016/j.cub.2018.04.015 29779873 PMC5988561

[52] Tataridas-Pallas N, Aman Y, Williams R, Chapman H, Cheng KJH, Gomez-Paredes C, et al. Mitochondrial clearance and increased HSF-1 activity are coupled to promote longevity in fasted *Caenorhabditis elegans*. iScience. 2024;27(6):109834. doi: 10.1016/j.isci.2024.109834 38784016 PMC11112483

[53] Stefana MI, Driscoll PC, Obata F, Pengelly AR, Newell CL, MacRae JI, et al. Developmental diet regulates *Drosophila* lifespan via lipid autotoxins. Nat Commun. 2017;8(1):1384. doi: 10.1038/s41467-017-01740-9 29123106 PMC5680271

[54] Dobson AJ, Ezcurra M, Flanagan CE, Summerfield AC, Piper MDW, Gems D, et al. Nutritional programming of lifespan by FOXO inhibition on sugar-rich diets. Cell Rep. 2017;18(2):299–306. doi: 10.1016/j.celrep.2016.12.029 28076775 PMC5263231

[55] Kosakamoto H, Obata F, Kuraishi J, Aikawa H, Okada R, Johnstone JN, et al. Early-adult methionine restriction reduces methionine sulfoxide and extends lifespan in *Drosophila*. Nat Commun. 2023;14(1):7832. doi: 10.1038/s41467-023-43550-2 38052797 PMC10698029

[56] Osmond C, Barker DJ, Winter PD, Fall CH, Simmonds SJ. Early growth and death from cardiovascular disease in women. BMJ. 1993;307(6918):1519–24. doi: 10.1136/bmj.307.6918.1519 8274920 PMC1679586

[57] Syddall HE, Aihie Sayer A, Dennison EM, Martin HJ, Barker DJP, Cooper C. Cohort profile: the Hertfordshire cohort study. Int J Epidemiol. 2005;34(6):1234–42. doi: 10.1093/ije/dyi127 15964908

[58] Kuh D, Ben-Shlomo Y, Lynch J, Hallqvist J, Power C. Life course epidemiology. J Epidemiol Community Health. 2003;57(10):778–83. doi: 10.1136/jech.57.10.778 14573579 PMC1732305

[59] Schulz LC. The Dutch Hunger Winter and the developmental origins of health and disease. Proc Natl Acad Sci U S A. 2010;107(39):16757–8. doi: 10.1073/pnas.1012911107 20855592 PMC2947916

[60] Thurner S, Klimek P, Szell M, Duftschmid G, Endel G, Kautzky-Willer A, et al. Quantification of excess risk for diabetes for those born in times of hunger, in an entire population of a nation, across a century. Proc Natl Acad Sci U S A. 2013;110(12):4703–7. doi: 10.1073/pnas.1215626110 23487754 PMC3607051

[61] Lumey LH, Khalangot MD, Vaiserman AM. Association between type 2 diabetes and prenatal exposure to the Ukraine famine of 1932-33: a retrospective cohort study. Lancet Diabetes Endocrinol. 2015;3(10):787–94. doi: 10.1016/S2213-8587(15)00279-X 26342852

[62] Finer S, Iqbal MS, Lowe R, Ogunkolade BW, Pervin S, Mathews C, et al. Is famine exposure during developmental life in rural Bangladesh associated with a metabolic and epigenetic signature in young adulthood? A historical cohort study. BMJ Open. 2016;6(11):e011768. doi: 10.1136/bmjopen-2016-011768 27881521 PMC5168545

[63] Abate KH, Arage G, Hassen H, Abafita J, Belachew T. Impact of prenatal famine exposure on adulthood fasting blood glucose level. Sci Rep. 2022;12(1):6198. doi: 10.1038/s41598-022-10120-3 35418574 PMC9008050

[64] Ma T, Hao XM, Zhang X, Liu XY, Wang YM, Zhang QS. In utero and childhood exposure to the great Chinese famine and risk of aging in adulthood. Sci Rep. 2024;14(1):25089. doi: 10.1038/s41598-024-77283-z 39443668 PMC11499915

[65] Tarry-Adkins JL, Ozanne SE. Nutrition in early life and age-associated diseases. Ageing Res Rev. 2017;39:96–105. doi: 10.1016/j.arr.2016.08.003 27594376

[66] Gracner T, Boone C, Gertler PJ. Exposure to sugar rationing in the first 1000 days of life protected against chronic disease. Science. 2024;386(6725):1043–8. doi: 10.1126/science.adn5421 39480913 PMC12238948

[67] Menting MD, Mintjens S, van de Beek C, Frick CJ, Ozanne SE, Limpens J, et al. Maternal obesity in pregnancy impacts offspring cardiometabolic health: systematic review and meta‐analysis of animal studies. Obes Rev. 2019;20(5):675–85. doi: 10.1111/obr.12817 30633422 PMC6849816

[68] Schoonejans JM, Ozanne SE. Developmental programming by maternal obesity: lessons from animal models. Diabet Med. 2021;38(12):e14694. doi: 10.1111/dme.14694 34553414

[69] Contreras RE, Schriever SC, Pfluger PT. Physiological and epigenetic features of yoyo dieting and weight control. Front Genet. 2019;10:1015. doi: 10.3389/fgene.2019.01015 31921275 PMC6917653

[70] Nordmo M, Danielsen YS, Nordmo M. The challenge of keeping it off, a descriptive systematic review of high-quality, follow-up studies of obesity treatments. Obes Rev. 2020;21(1):e12949. doi: 10.1111/obr.12949 31675146

[71] Hinte LC, Castellano-Castillo D, Ghosh A, Melrose K, Gasser E, Noé F, et al. Adipose tissue retains an epigenetic memory of obesity after weight loss. Nature. 2024;636(8042):457–65. doi: 10.1038/s41586-024-08165-7 39558077 PMC11634781

[72] Schmitz J, Evers N, Awazawa M, Nicholls HT, Brönneke HS, Dietrich A, et al. Obesogenic memory can confer long-term increases in adipose tissue but not liver inflammation and insulin resistance after weight loss. Mol Metab. 2016;5(5):328–39. doi: 10.1016/j.molmet.2015.12.001 27110485 PMC4837291

[73] Dong H, Sun Y, Nie L, Cui A, Zhao P, Leung WK, et al. Metabolic memory: mechanisms and diseases. Signal Transduct Target Ther. 2024;9(1):38. doi: 10.1038/s41392-024-01755-x 38413567 PMC10899265

[74] Trim W, Turner JE, Thompson D. Parallels in immunometabolic adipose tissue dysfunction with ageing and obesity. Front Immunol. 2018;9:169. doi: 10.3389/fimmu.2018.00169 29479350 PMC5811473

[75] Ravussin E, Redman LM, Rochon J, Das SK, Fontana L, Kraus WE, et al. A 2-year randomized controlled trial of human caloric restriction: feasibility and effects on predictors of health span and longevity. J Gerontol A Biol Sci Med Sci. 2015;70(9):1097–104. doi: 10.1093/gerona/glv057 26187233 PMC4841173

[76] Huffman KM, Parker DC, Bhapkar M, Racette SB, Martin CK, Redman LM, et al. Calorie restriction improves lipid-related emerging cardiometabolic risk factors in healthy adults without obesity: distinct influences of BMI and sex from CALERIE™ a multicentre, phase 2, randomised controlled trial. EClinicalMedicine. 2022;43:101261. doi: 10.1016/j.eclinm.2021.101261 35028547 PMC8741476

[77] Das SK. Legacy effects of CALERIE^TM^, a 2-year calorie restriction intervention, on hallmarks of healthspan and aging [Clinical trial registration] [Internet]. clinicaltrials.gov; 2025 Oct [cited 2025 Dec 10]. Report No.: NCT05651620. Available from: https://clinicaltrials.gov/study/NCT05651620

[78] Pal S, Tyler JK. Epigenetics and aging. Sci Adv. 2016;2(7):e1600584. doi: 10.1126/sciadv.1600584 27482540 PMC4966880

[79] Verma A, Lindroth AM. The emerging intertwined activities of metabolism and epigenetics unveils culprits and prospects in cancer. Exp Mol Med. 2025;57(9):1928–39. doi: 10.1038/s12276-025-01537-7 40935853 PMC12508213

[80] Kim M, Costello J. DNA methylation: an epigenetic mark of cellular memory. Exp Mol Med. 2017;49(4):e322. doi: 10.1038/emm.2017.10 28450738 PMC6130213

[81] Greer EL, Maures TJ, Ucar D, Hauswirth AG, Mancini E, Lim JP, et al. Transgenerational epigenetic inheritance of longevity in *Caenorhabditis elegans*. Nature. 2011;479(7373):365–71. doi: 10.1038/nature10572 22012258 PMC3368121

[82] Moore RS, Kaletsky R, Murphy CT. Piwi/PRG-1 argonaute and TGF-β mediate transgenerational learned pathogenic avoidance. Cell. 2019;177(7):1827-1841.e12. doi: 10.1016/j.cell.2019.05.024 31178117 PMC7518193

[83] Perez MF, Lehner B. Intergenerational and transgenerational epigenetic inheritance in animals. Nat Cell Biol. 2019;21(2):143–51. doi: 10.1038/s41556-018-0242-9 30602724

[84] Kaletsky R, Moore RS, Vrla GD, Parsons LR, Gitai Z, Murphy CT. C. elegans interprets bacterial non-coding RNAs to learn pathogenic avoidance. Nature. 2020;586(7829):445–51. doi: 10.1038/s41586-020-2699-5 32908307 PMC8547118

[85] Al-Refaie N, Padovani F, Hornung J, Pudelko L, Binando F, Del Carmen Fabregat A, et al. Fasting shapes chromatin architecture through an mTOR/RNA Pol I axis. Nat Cell Biol. 2024;26(11):1903–17. doi: 10.1038/s41556-024-01512-w 39300311 PMC11567895

[86] Bungard D, Fuerth BJ, Zeng P-Y, Faubert B, Maas NL, Viollet B, et al. Signaling kinase AMPK activates stress-promoted transcription via histone H2B phosphorylation. Science. 2010;329(5996):1201–5. doi: 10.1126/science.1191241 20647423 PMC3922052

[87] Singh A, Kumar N, Matai L, Jain V, Garg A, Mukhopadhyay A. A chromatin modifier integrates insulin/IGF-1 signalling and dietary restriction to regulate longevity. Aging Cell. 2016;15(4):694–705. doi: 10.1111/acel.12477 27039057 PMC4933660

[88] Li X, Mei Q, Yu Q, Wang M, He F, Xiao D. The TORC1 activates Rpd3L complex to deacetylate Ino80 and H2A.Z and repress autophagy. Sci Adv. 2023;9(10):eade8312. doi: 10.1126/sciadv.ade8312 PMC9995077 36888706

[89] Besson D, Vaur S, Vazquez S, Tournier S, Gachet Y, Birot A. Interplay between cohesin and TORC1 links chromosome segregation and gene expression to environmental changes. eLife. 2025. doi: 10.7554/eLife.108275.1 PMC13225845 42223017

[90] Iqbal F. AMPK regulates chromatin dynamics through lamina. Biophys J. 2024;123(3):367a. doi: 10.1016/j.bpj.2023.11.2238

[91] Sun G, Leclerc GJ, Chahar S, Barredo JC. AMPK associates with chromatin and phosphorylates the TAF-1 subunit of the transcription initiation complex to regulate histone gene expression in ALL cells. Mol Cancer Res. 2023;21(12):1261–73. doi: 10.1158/1541-7786.MCR-23-0502 37682252 PMC10690046

[92] Riedel CG, Dowen RH, Lourenco GF, Kirienko NV, Heimbucher T, West JA, et al. DAF-16 employs the chromatin remodeller SWI/SNF to promote stress resistance and longevity. Nat Cell Biol. 2013;15(5):491–501. doi: 10.1038/ncb2720 23604319 PMC3748955

[93] Maybury-Lewis SY, Brown AK, Yeary M, Sloutskin A, Dhakal S, Juven-Gershon T, et al. Changing and stable chromatin accessibility supports transcriptional overhaul during neural stem cell activation and is altered with age. Aging Cell. 2021;20(11):e13499. doi: 10.1111/acel.13499 34687484 PMC8590101

[94] Audesse AJ, Dhakal S, Hassell L-A, Gardell Z, Nemtsova Y, Webb AE. FOXO3 directly regulates an autophagy network to functionally regulate proteostasis in adult neural stem cells. PLoS Genet. 2019;15(4):e1008097. doi: 10.1371/journal.pgen.1008097 30973875 PMC6478346

[95] Bianco-Miotto T, Craig JM, Gasser YP, van Dijk SJ, Ozanne SE. Epigenetics and DOHaD: from basics to birth and beyond. J Dev Orig Health Dis. 2017;8(5):513–9. doi: 10.1017/S2040174417000733 28889823

[96] Fazeli SA, Soleimani Samarkhazan H. Metabolic memory following metabolic bariatric surgery: mechanisms, clinical implications, and strategies for long-term success. Obes Surg. 2025;35(12):5606–24. doi: 10.1007/s11695-025-08417-z 41310268

[97] Bheda P. Metabolic transcriptional memory. Mol Metab. 2020;38:100955. doi: 10.1016/j.molmet.2020.01.019 32240621 PMC7300383

[98] Ni Z, Ebata A, Alipanahiramandi E, Lee SS. Two SET domain containing genes link epigenetic changes and aging in *Caenorhabditis elegans*. Aging Cell. 2012;11(2):315–25. doi: 10.1111/j.1474-9726.2011.00785.x 22212395 PMC3306474

[99] Lu YX, Regan JC, Eßer J, Drews LF, Weinseis T, Stinn J, et al. A TORC1-histone axis regulates chromatin organisation and non-canonical induction of autophagy to ameliorate ageing. Elife. 2021;10:e62233. doi: 10.7554/eLife.62233 33988501 PMC8186904

[100] Johnson AA, Akman K, Calimport SRG, Wuttke D, Stolzing A, de Magalhães JP. The role of DNA methylation in aging, rejuvenation, and age-related disease. Rejuvenation Res. 2012;15(5):483–94. doi: 10.1089/rej.2012.1324 23098078 PMC3482848

[101] Ma Z, Wang H, Cai Y, Wang H, Niu K, Wu X, et al. Epigenetic drift of H3K27me3 in aging links glycolysis to healthy longevity in *Drosophila*. Kaeberlein M, editor. eLife. 2018;7:e35368. doi:10.7554/eLife.35368 10.7554/eLife.35368 PMC5991832 29809154

[102] Reichert P, Caudron F. Mnemons and the memorization of past signaling events. Curr Opin Cell Biol. 2021;69:127–35. doi: 10.1016/j.ceb.2021.01.005 33618243

[103] Zgutka K, Tkacz M, Tomasiak P, Tarnowski M. A role for advanced glycation end products in molecular ageing. Int J Mol Sci. 2023;24(12):9881. doi: 10.3390/ijms24129881 37373042 PMC10298716

[104] Giorgi C, Marchi S, Simoes ICM, Ren Z, Morciano G, Perrone M, et al. Mitochondria and reactive oxygen species in aging and age-related diseases. Int Rev Cell Mol Biol. 2018;340:209–344. doi: 10.1016/bs.ircmb.2018.05.006 30072092 PMC8127332

[105] O’Donovan A, Pantell MS, Puterman E, Dhabhar FS, Blackburn EH, Yaffe K, et al. Cumulative inflammatory load is associated with short leukocyte telomere length in the Health, Aging and Body Composition Study. PLoS One. 2011;6(5):e19687. doi: 10.1371/journal.pone.0019687 21602933 PMC3094351

[106] Kim H, Worsley O, Yang E, Purbojati RW, Liang AL, Tan W, et al. Persistent changes in liver methylation and microbiome composition following reversal of diet-induced non-alcoholic-fatty liver disease. Cell Mol Life Sci. 2019;76(21):4341–54. doi: 10.1007/s00018-019-03114-4 31119300 PMC11105172

[107] Obata F, Fons CO, Gould AP. Early-life exposure to low-dose oxidants can increase longevity via microbiome remodelling in *Drosophila*. Nat Commun. 2018;9(1):975. doi: 10.1038/s41467-018-03070-w 29515102 PMC5841413

[108] Lynn MA, Eden G, Ryan FJ, Bensalem J, Wang X, Blake SJ, et al. The composition of the gut microbiota following early-life antibiotic exposure affects host health and longevity in later life. Cell Rep. 2021;36(8):109564. doi: 10.1016/j.celrep.2021.109564 34433065

[109] Siersbæk M, Varticovski L, Yang S, Baek S, Nielsen R, Mandrup S. High fat diet-induced changes of mouse hepatic transcription and enhancer activity can be reversed by subsequent weight loss. Sci Rep. 2017;7(1):40220. doi: 10.1038/srep40220 28071704 PMC5223143

[110] Cottam MA, Caslin HL, Winn NC, Hasty AH. Multiomics reveals persistence of obesity-associated immune cell phenotypes in adipose tissue during weight loss and weight regain in mice. Nat Commun. 2022;13:2950. doi: 10.1038/s41467-022-30646-4 35618862 PMC9135744

[111] Miranda AMA, McAllan L, Mazzei G, Andrew I, Davies I, Ertugrul M, et al. Selective remodelling of the adipose niche in obesity and weight loss. Nature. 2025;644(8077):769–79. doi: 10.1038/s41586-025-09233-2 40634602 PMC12367556

[112] Martyniak K, Masternak MM. Changes in adipose tissue cellular composition during obesity and aging as a cause of metabolic dysregulation. Exp Gerontol. 2017;94:59–63. doi: 10.1016/j.exger.2016.12.007 27939445 PMC5466486

[113] Du N, Song L, Yang R, Liu K, Niu Z, Zhang Z. Early-stage administration of hydroxytyrosol extends lifespan and delays aging in *C. elegans*. Biol Direct. 2025;20:62. doi: 10.1186/s13062-025-00634-x 40399944 PMC12096744

[114] Villareal DT. Obesity and accelerated aging. J Nutr Health Aging. 2023;27(5):312–3. doi: 10.1007/s12603-023-1922-0 37248754 PMC10349370

[115] Le Couteur DG, Raubenheimer D, Solon-Biet S, de Cabo R, Simpson SJ. Does diet influence aging? Evidence from animal studies. J Intern Med. 2024;295(4):400–15. doi: 10.1111/joim.13530 35701180 PMC12023453

[116] Dearden L, Ozanne SE. Early life impacts of maternal obesity: a window of opportunity to improve the health of two generations. Philos Trans R Soc Lond B Biol Sci. 2023;378(1885):20220222. doi: 10.1098/rstb.2022.0222 37482780 PMC10363703

[117] Brennan SL, Henry MJ, Nicholson GC, Kotowicz MA, Pasco JA. Socioeconomic status and risk factors for obesity and metabolic disorders in a population-based sample of adult females. Prev Med. 2009;49(2–3):165–71. doi: 10.1016/j.ypmed.2009.06.021 19576925

[118] Blanquet M, Legrand A, Pélissier A, Mourgues C. Socio-economics status and metabolic syndrome: a meta-analysis. Diabetes Metab Syndr. 2019;13(3):1805–12. doi: 10.1016/j.dsx.2019.04.003 31235098

[119] Wells JC, Sawaya AL, Wibaek R, Mwangome M, Poullas MS, Yajnik CS, et al. The double burden of malnutrition: aetiological pathways and consequences for health. The Lancet. 2020;395(10217):75–88. doi: 10.1016/s0140-6736(19)32472-9 PMC7613491 31852605

[120] Kivimäki M, Pentti J, Frank P, Liu F, Blake A, Nyberg ST, et al. Social disadvantage accelerates aging. Nat Med. 2025;31(5):1635–43. doi: 10.1038/s41591-025-03563-4 40087516 PMC12092251

[121] Jiang N, Cheng CJ, Liu Q, Strong R, Gelfond J, Nelson JF. Deciphering the timing and impact of life-extending interventions: temporal efficacy profiler distinguishes early, midlife, and senescence phase efficacies. Nat Commun. 2025;16(1):10164. doi: 10.1038/s41467-025-65158-4 41258280 PMC12630856

[122] Coupe N, Cotterill S, Peters S. Tailoring lifestyle interventions to low socio-economic populations: a qualitative study. BMC Public Health. 2018;18(1):967. doi: 10.1186/s12889-018-5877-8 30075716 PMC6076398

